# Prognostic value of transcranial facial nerve motor-evoked potentials in predicting facial nerve function following cerebellopontine angle tumorectomy

**DOI:** 10.1097/MD.0000000000012576

**Published:** 2018-10-05

**Authors:** Hongmei Song, Chengyuan Ma, Dahai Xu, Mingxin Yu, Jiachun Feng, Lichao Sun

**Affiliations:** a Department of Neurosurgery; b Department of Emergency Medicine; c Department of Neurology, The First Hospital of Jilin University, Changchun, Jilin 130021, China.

**Keywords:** cerebellopontine angle, facial nerve motor-evoked potentials, facial nerve paralysis, prognosis, transcranial electrical stimulation, tumorectomy

## Abstract

Facial nerve paralysis is a common complication following cerebellopontine angle (CPA) surgery. This study investigated the prognostic value of facial nerve motor-evoked potentials (FNMEPs) elicited by transcranial electrical stimulation for facial nerve outcome after CPA tumorectomy.

A total of 95 patients were enrolled in this study between January 2014 and January 2016. All these patients underwent CPA tumorectomy (unilateral, n = 95; bilateral, n = 1). Intraoperative FNMEP elicited by transcranial electrical stimulation was recorded. The short- and long-term postoperative facial nerve functions were evaluated according to the House–Brackmann (HB) scale. The correlation between perioperative changes in the FNMEP stimulus threshold (delta FNMEP = postoperative stimulus threshold level–preoperative stimulus threshold level) and postoperative facial nerve functions were analyzed.

On the first day postoperatively, the facial nerve function was HB grade I in 67, grade II in 17, grade III in 7, and grade IV in 5 facial nerves. One year postoperatively, the facial nerve function was grade I in 80, grade II in 11, grade III in 3, and grade IV in 2 facial nerves. The delta FNMEP was significantly correlated with the short- and long-term facial nerve function; receiver operating characteristic (ROC) curves yielded a cut-off delta FNMEP value of 30 V (sensitivity, 91.3%; specificity, 98.6%) and 75 V (sensitivity, 100%; specificity, 98.8%) for predicting short- and long-term facial nerve function damage, respectively.

FNMEP elicited by transcranial electrical stimulation is an effective and safe approach for predicting facial nerve function in CPA tumorectomy. A high delta FNMEP is a potential indicator for the prediction of postoperative facial nerve damage.

## 1 Introduction

Cerebellopontine angle (CPA) neoplasms comprise approximately 10% of all intracranial tumors.^[1]^ Surgical resection is the mainstream therapeutic option for these entities, and cranial nerve damage is a common complication following CPA surgery.^[2,3]^ Patients may develop facial nerve paralysis immediately after surgery or may develop an early delayed postoperative paralysis, which severely impairs the individual's quality of life. Currently, various modalities of intraoperative facial nerve mapping and monitoring have been developed for the anatomical and functional preservation of the facial nerve.^[4–9]^ Direct electrical stimulation of the facial nerve is the conventional approach for intraoperative electrophysiological mapping.^[10,11]^ During CPA tumorectomy, surgeons stimulate the exposed facial nerve, and the evoked compound muscle action potential (CMAP) in muscles dominated by the facial nerve can be recorded. However, this CMAP can only represent the anatomical and functional integrity of the nerve segment between the stimulation site and the recording electrode. Additionally, when the facial nerve was enveloped by tumor tissues, direct electrical stimulation may be impossible. Recently, a facial nerve motor-evoked potential (FNMEP) elicited by transcranial electrical stimulation has been used in CPA surgeries.^[12–15]^ Transcranial FNMEP is a non-invasive monitoring technique, in which scalp electrodes stimulate the facial nerve-corresponding motor cortex triggering CMAP in the downstream muscles. An unattenuated waveform appearing on the target muscle group represents that the facial nerve pathway and function are intact. Some scholars recommend the amplitude reduction as an indicator predicting facial nerve damage, while the quantitative alarm criteria have yet to be clearly established.^[16,17]^

In this study, we investigated the prognostic value of FNMEP elicited by transcranial electrical stimulation for predicting facial nerve outcome following CPA tumorectomy.

## 2 Patient and methods

### 2.1 Patients

A total of 95 patients with CPA tumor(s) were enrolled in this study between January 2014 and January 2016. All these patients underwent CPA tumorectomy (unilateral, n = 95; bilateral, n = 1) via a retrosigmoid approach. The exclusion criteria included: epilepsy, or preoperative facial nerve paralysis.

### 2.2 Assessment of facial nerve function

The perioperative and follow-up facial nerve functions were evaluated according to the House–Brackmann (HB) Facial Nerve Grading System^[18]^: grade I, normal; grade II, mild facial nerve dysfunction; grade III, moderate facial nerve dysfunction; grade IV, moderate-severe facial nerve dysfunction; grade V, severe facial nerve dysfunction; and grade VI, complete paralysis of the facial nerve. The short-term facial nerve outcome was defined as facial nerve function on the first day after surgery, and the long-term facial nerve outcome was defined as facial nerve function 1 year postoperatively. Based on the HB grading, the patients were classified into 2 groups: those with preserved facial nerve function (HB grades I and II) and those with facial nerve dysfunction (HB grade ≥III).

### 2.3 Intraoperative electrophysiological monitoring

**Figure 1:**
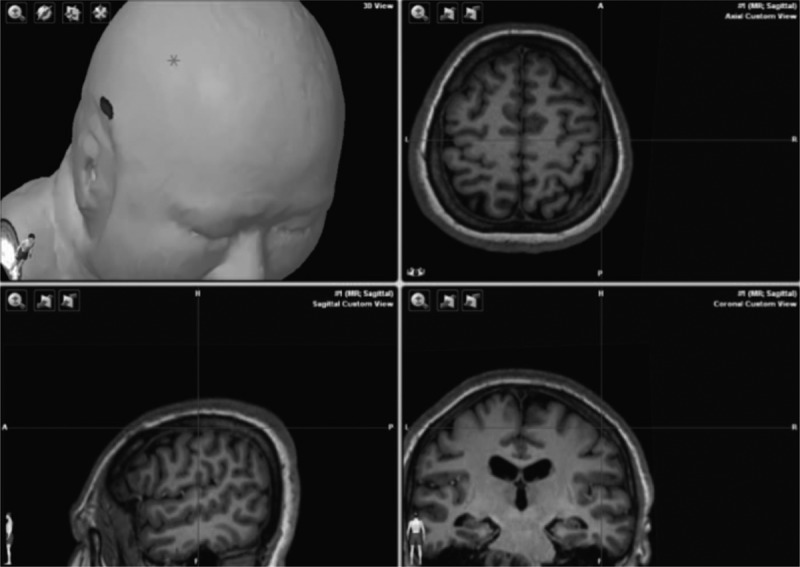
Localization of primary facial nerve motor cortex, the primary facial nerve motor cortex was localized using intraoperative neuronavigator.

**Figure 2:**
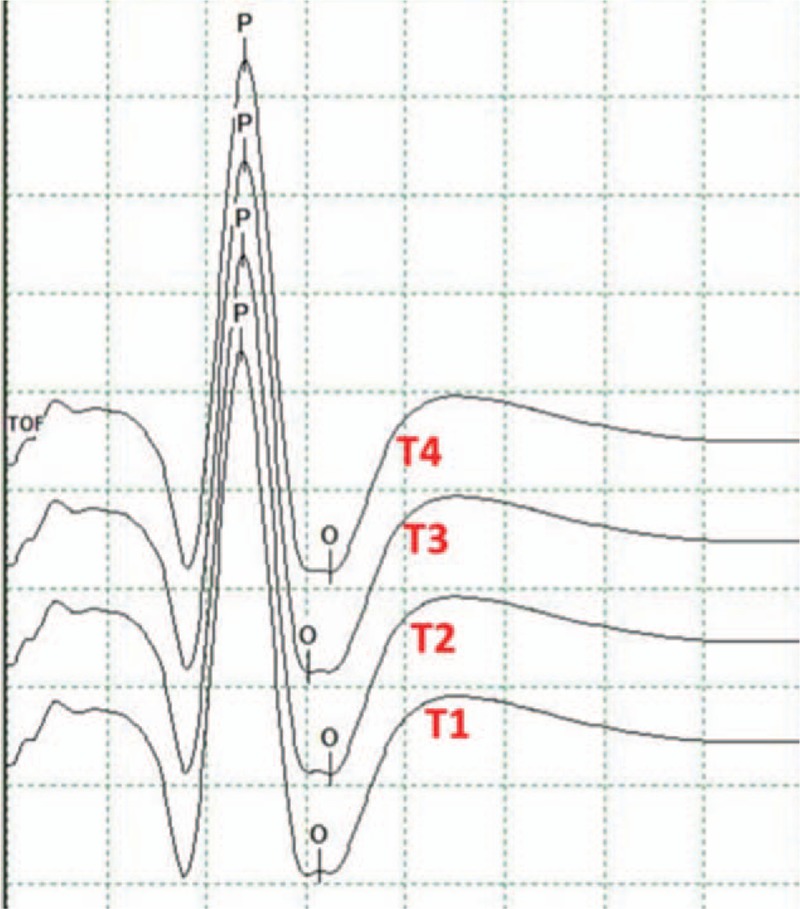
Evaluation of muscle relaxant metabolism, the train-of-four (TOF) value was used to monitor the neuromuscular blockade and to evaluate the metabolism of muscle relaxant. A ratio T4/T1 of 1.0 indicated that the muscle relaxant was completely metabolized.

General anesthesia was induced with a constant infusion of remifentanil and propofol and a single-dose usage of short-term muscle relaxant. With the patient in the proper position (lateral decubitus position in most cases), intraoperative electrophysiological monitoring was performed by an experienced group to record the somatosensory-evoked potentials, brainstem auditory-evoked potentials, and CMAPs in muscles dominated by cranial nerves (trigeminal nerve, facial nerve, and lower cranial nerves). A pair of subcutaneous needle electrodes were placed overlying the facial nerve-dominating mentalis for monitoring the CMAPs, and these electrodes were also used to record the FNMEP elicited by transcranial electrical stimulation. A transcranial electrical stimulation (32Xltek, Natus, California) was delivered by placing an anode at the primary facial nerve motor cortex contralateral to the tumor; the facial nerve motor cortex was localized with an intraoperative neuronavigation system (Fig. 1). We used the train-of-four value to monitor the neuromuscular blockade and to evaluate the metabolism of muscle relaxant (Fig. 2). FNMEP elicited by transcranial electrical stimulation was documented every 5 to 10 minutes throughout the surgical procedure. We selected the mentalis as the target muscle, since the mentalis has the longest distance from the stimulus point, which can minimize the stimulus-related artifacts. A train of two-pulse (initial interstimulus interval = 2 ms) transcranial electrical stimulation was used. Each pulse lasted for 50 μs; the bandpass filter was set at 150 to 3000 Hz. The stimulus threshold was set as the criterion recommended by Calancie in 2008^[19]^: evoked muscle response that exceeded 20 μV in peak-to-peak amplitude, and which had an appropriate response latency (latency to mentalis was about 15–20 ms) was considered an effective response. After the muscle relaxant was completely metabolized, an initial stimulus intensity at 50 V was used, increasing by 1-V increments until the valid FNMEP in the target mentalis could be recorded (the maximal stimulus intensity was 500 V). The real-time stimulus intensity was defined as the stimulus threshold baseline. When the dura mater was incised, the stimulus threshold was recorded as the preoperative FNMEP stimulus threshold level; after the tumorectomy was finished, the stimulus threshold was recorded as the postoperative FNMEP stimulus threshold level. The perioperative change in the FNMEP stimulus threshold (delta FNMEP) was calculated: delta FNMEP = postoperative stimulus threshold level – preoperative stimulus threshold level. Additionally, another pair of subcutaneous needle electrodes was placed overlying the contralateral abductor pollicis brevis for recording the upper-limb MEP, guaranteeing that the transcranial electrical stimulation stimulated the tractus corticobulbaris rather than the peripheral tractus corticospinalis. During the tumorectomy, if any stimulus threshold changes were noticed, we would inform the operators and they suspended the surgical procedures until the stimulus threshold recovered. Eventually, the stimulus threshold after tumorectomy was compared to the stimulus threshold when the dura mater was incised, and the associations between stimulus threshold changes and postoperative facial nerve functional outcomes were evaluated.

### 2.4 Statistical analysis

SPSS 24.0 software (IBM SPSS, Armonk, NY) was used for statistical analyses. Spearman correlation analysis was used to analyze the correlation between perioperative FNMEP changes (delta FNMEP = postoperative level – preoperative level) and facial nerve function grading. Mann–Whitney *U* test was used to compare the difference in delta FNMEP between the preserved facial nerve function group and the damaged facial nerve function group. A receiver operating characteristic (ROC) curve was used to calculate the cut-off value of delta FNMEP for predicting facial nerve function damage. *P*-values ≤.05 were considered to indicate a statistically significant significance.

## 3 Results

### 3.1 Demographic data

The study population included 66 males and 29 females, with an average age of 51.4 ± 11.4 years. Fifty-six patients had a CPA tumor on the left side, 38 patients had a CPA tumor on the right side, and 1 patient had bilateral CPA tumors. The histopathological patterns included acoustic schwannoma (n = 52), meningioma (n = 32), and epidermoidoma (n = 11). The functions of 96 facial nerves were monitored. There were no electrophysiological monitoring-related complications.

### 3.2 Facial nerve function

On the first day postoperatively, the facial nerve function was HB grade I in 67 (69.8%), grade II in 17 (17.7%), grade III in 7 (7.3%), and grade IV in 5 (5.2%) facial nerves. One year postoperatively, the facial nerve function was HB grade I in 80 (83.3%), grade II in 11 (11.5%), grade III in 3 (3.1%), and grade IV in 2 (2.1%) facial nerves.

### 3.3 Correlation between FNMEP stimulus threshold changes and facial nerve functions

**Figure 3:**
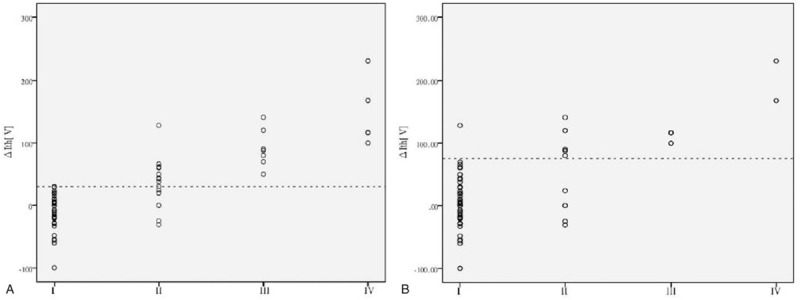
Scatter diagram showing the correlation between the delta facial nerve motor-evoked potential (FNMEP) and the facial nerve functions, (A) the delta FNMEPs (ΔIth) were positively correlated with the short-term facial nerve functions (rho = 0.645, *P* < .001). The dotted line indicates a delta FNMEP of 30 V. (B) The delta FNMEPs (ΔIth) were positively correlated with the long-term facial nerve functions (rho = 0.375, *P* < .001). The dotted line indicates a delta FNMEP of 75 V.

**Figure 4:**
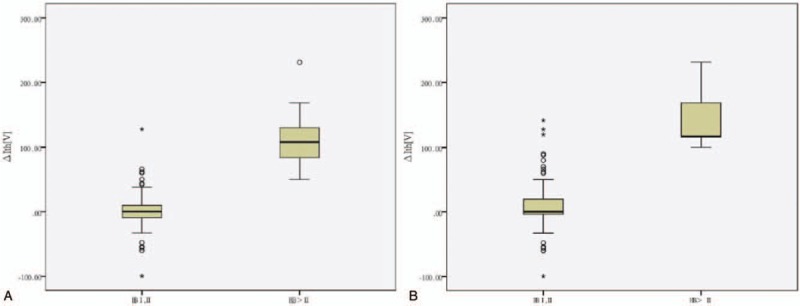
Comparison of delta facial nerve motor-evoked potential (FNMEP) between the facial nerve function reservation group and the facial nerve function damage group, (A) based short-term facial nerve function assessment, the delta FNMEP in the facial nerve function reservation group (n = 84) was significantly lower than those in the facial nerve function damage group (n = 12) (*P* < .001). (B) Based long-term facial nerve function assessment, the delta FNMEP in the facial nerve function reservation group (n = 92) was also significantly lower than those in the facial nerve function damage group (n = 5) (*P* < .001).

Spearman correlation analysis showed the delta FNMEP was positively correlated with the short-term facial nerve function grades (rho = 0.645, *P* < .001) as well as the long-term facial nerve function grades (rho = 0.375, *P* < .001). The scatter diagrams are shown in Figure 3. The Mann–Whitney *U* test showed a significant difference in the delta FNMEP between the preserved facial nerve function group and the damaged facial nerve function group classified according to either short-term or long-term facial nerve function assessment (both *P* < .001; Fig. 4).

### 3.4 Alert cut-off values

**Figure 5:**
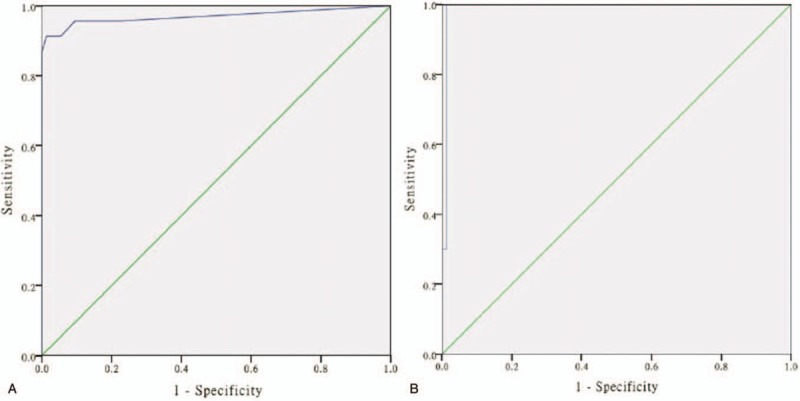
ROC curve analyses for short-term andlong-termfacial nerve function damage, (A) ROC curve analysis yielded a cut-off delta FNMEP value of 30 V (sensitivity, 91.3%; specificity, 98.6%) for predicting short-term facial nerve function damage. The AUC is 0.976 (95% confidence interval: 0.936–1.000; Youden index: 0.899). (B) ROC curve analysis yielded a cut-off value of 75 V (sensitivity, 100%; specificity, 98.8%) for predicting long-term facial nerve function damage. The AUC is 0.992 (95% confidence interval: 0.975–1.000; Youden index: 0.988). AUC = area under the curve, FNMEP = facial nerve motor-evoked potential, ROC = receiver operating characteristic.

ROC curve analyses yielded a cut-off delta FNMEP value of 30 V (sensitivity, 91.3%; specificity, 98.6%) for predicting short-term facial nerve function damage (Fig. 5A) and a cut-off value of 75 V (sensitivity, 100%; specificity, 98.8%) for predicting long-term facial nerve function damage (Fig. 5B).

## 4 Illustrative case

**Figure 6:**
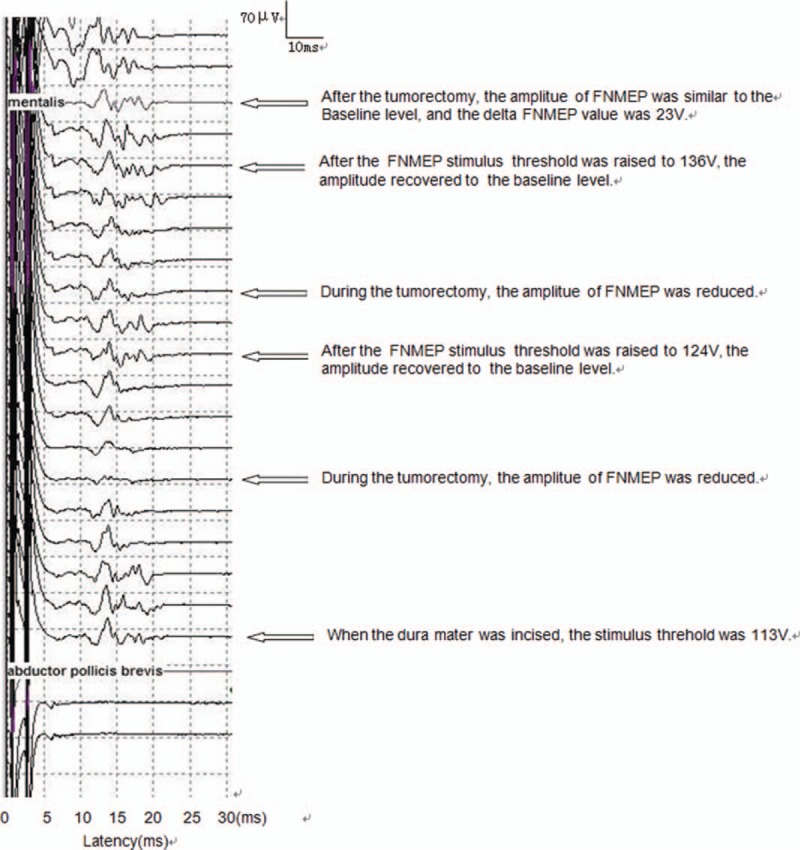
Dynamic monitoring results in the illustrative case.

A 74-year-old woman was diagnosed with meningioma in the right CPA region. The preoperative facial nerve function was HB grade I. When the dura mater was incised, the FNMEP stimulus threshold was 113 V. Intraoperatively, the response amplitude was significantly reduced, and the FNMEP stimulus threshold needed to be raised. After the tumor was completely removed, the FNMEP stimulus threshold was 136 V, yielding a delta FNMEP value of 23 V. The short- and long-term facial nerve function evaluations were both HB grade I. The dynamic monitoring results are presented in Figure 6.

## 5 Discussion

In the current study, we used a “threshold-level” method for intraoperative monitoring of facial nerve function, and we recorded the contralateral stimulus threshold levels as a normal control. We found the cut-off value of delta FNMEP for predicting long-term facial nerve outcome was larger than that for predicting short-term facial nerve function, suggesting that a higher intraoperative delta FNMEP may be associated with facial nerve damage requiring a long-term recovery. Cosetti et al also used the delta FNMEP as a quantitative criterion during CPA tumor resection, and they found that patients with HB I or II at a mean 10-month follow-up had an average delta FNMEP of 0.04 V, whereas those with HB >2 had an average delta FNMEP of 57 V.^[4]^ Bozinov et al proposed that a threshold increase of >20 mA for eliciting the FNMEP may be a warning criterion predicting postoperative impaired facial nerve function.^[20]^

An FNMEP elicited by transcranial electrical stimulation was first reported by Merton and Morton in 1980,^[21]^ and the FNMEP was applied in tumorectomy by Zhou et al in 2001.^[22]^ This monitoring approach records the CMAP in the target muscle (mentalis) evoked by stimulating the facial nerve motor cortex.^[23]^ Transcranial electrical stimulation is less invasive than conventional direct electronical stimulation, and the FNMEP is considered to be closely associated with the postoperative facial nerve function. In CPA tumorectomy, transcranial FNMEP can be applied for real-time evaluation of the facial nerve function, facilitating the early identification of facial nerve damage and promoting maximal safe resection of the CPA tumors with facial nerve reservation.^[22,24]^ The FNMEP has significant superiority when the tumor is giant in size and envelops the facial nerve. Notably, transcranial FNMEP may be more sensitive than other traditional parameters, and evaluation using a stimulus threshold is a quantitative method; when the stimulus threshold level is increased, surgeons should be alert to the potential for facial nerve damage. Furthermore, transcranial FNMEP is much safer than conservative continuous electromyography. Although transcranial magnetic stimulation is non-invasive and painfree and it can stimulate the proximal facial nerve, magnetic stimulation does not have a fixed stimulation site; electrical stimulation has a better spatial resolution as well as a superior temporal resolution.

Electromyography activity occurs only at the time when the facial nerve is irritated intraoperatively, while the absence of electromyography activity may indicate a structurally and functionally integrated facial nerve or total loss of facial nerve function.^[19]^ Romstöck et al found continuous EMG monitoring could reduce the rate of facial nerve injury following CPA surgery.^[25]^ Free-running EMG is applicable for all facial muscles, however, FMEP is less well controlled at the upper parts of the face.^[26]^

Various factors may affect the monitoring of motor-evoked potentials, such as administration of anesthetic muscle relaxants, numbers of recording electrodes, and individual physical differences. The causes of postoperative facial nerve paralysis may also be variable, including transient conduction block of facial motor nerve, neurapraxia, mechanical axonotmesis, edema or ischemia-hypoxia of facial nerve, and herpesvirus activation. In the current study, we used the train-of-four value to evaluate the neuromuscular blockade and the metabolism of muscle relaxant, the relevant confounding factors being excluded.

The alarm criteria of FNMEP have yet to be established. Some scholars recommend a >50% reduction in amplitude as an indicator for predicting facial nerve damage.^[16,17]^ The amplitudes of FNMEP are heterogeneous and exact quantitative measurements are usually challenging; additionally, the “amplitude-warning” method requires a much stronger stimulus intensity, which may cause neck movement of the anesthetized patient and affect the surgical procedures. A recent study showed that in transcranial FNMEP, monitoring only 20% of motor neurons distributed in the target muscles can form potentials.^[20]^ We speculate that the amplitude of FNMEP may simply represent the function of partial neurons; when these neurons are not involved in facial nerve damage, the patient may develop facial palsy while the amplitude of FNMEP can be normal.

The current study also has some inherent limitations. The cut-off threshold level may lead to false negative prediction. ROC curve analysis yielded a cut-off value of 30 V for predicting short-term facial nerve outcome. However, in our cohort, 2 patients developed postoperative facial nerve paralysis in whom the intraoperative delta FNMEP was less than 30 V. Additionally, in some patients, the delta FNMEP was negative, which may be associated with the anesthesia anabiosis when the final FNMEP stimulus threshold was recorded. ROC curve analyses also produced a cut-off value of 70 V for predicting long-term facial nerve outcome. A long-term recovery can alleviate the nerve edema and facilitate nerve regeneration and reconstruction.

The safety of transcranial FNMEP monitoring is another important issue. MacDonald reviewed the clinical experience with intraoperative transcranial electrical stimulation motor-evoked potential monitoring in more than 15,000 cases; they found only 43 patients developed complications including tongue or lip laceration, cardiac arrhythmia, and intraoperative awareness.^[27]^ In our study, no electrophysiological monitoring-related complications were noted. We recommend transcranial FNMEP as a safe approach for intraoperative monitoring of facial nerve function.

In short, CPA tumors are associated with complex anatomy and the surgery is extremely challenging. Although intraoperative electrophysiological monitoring has been found valuable for evaluating and protecting the facial nerve function, proficient, and experienced surgical and electrophysiological skills are still fundamental and should be much more highlighted.

## 6 Conclusions

CPA tumorectomy is a challenging and delicate surgical procedure due to the complicated local anatomical structures. The FNMEP elicited by transcranial electrical stimulation is an effective and safe approach for predicting the short-term and long-term facial nerve function following CPA tumorectomy. A high delta FNMEP is a potential indicator for the prediction of postoperative facial nerve damage.

## Author contributions

**Data curation:** Chengyuan Ma.

**Formal analysis:** Dahai Xu.

**Investigation:** Hongmei Song.

**Methodology:** Jiachun Feng, Lichao Sun.

**Writing – original draft:** Mingxin Yu, Lichao Sun.

**Writing – review & editing:** Mingxin Yu, Lichao Sun.

## References

[1] Mahboubi H Haidar YM Moshtaghi O Postoperative complications and readmission rates following surgery for cerebellopontine angle schwannomas. Otol Neurotol 2016;37:1423–7. 27525710 10.1097/MAO.0000000000001178 PMC5929100

[2] Simon MV Neurophysiologic intraoperative monitoring of the vestibulocochlear nerve. J Clin Neurophysiol 2011;28:566–81. 22146352 10.1097/WNP.0b013e31823da494

[3] Prasad S Kamerer DB Hirsch BE Preservation of vestibular nerves in surgery of the cerebellopontine angle: effect on hearing and balance function. Am J Otolaryngol 1993;14:15–20. 8434714 10.1016/0196-0709(93)90004-q

[4] Cosetti MK Ming X Andrew R Intraoperative transcranial motor-evoked potential monitoring of the facial nerve during cerebellopontine angle tumor resection. J Neurol Surg Part B Skull Base 2012;73:308–15. 10.1055/s-0032-1321507 PMC3578638 24083121

[5] Tsutsui S Yamada H Hashizume H Quantification of the proportion of motor neurons recruited by transcranial electrical stimulation during intraoperative motor evoked potential monitoring. J Clin Monit Comput 2013;27:633–7. 23748599 10.1007/s10877-013-9480-3

[6] Joo BE Park SK Cho KR Real-time intraoperative monitoring of brainstem auditory evoked potentials during microvascular decompression for hemifacial spasm. J Neurosurg 2016;125:1061–7. 26824371 10.3171/2015.10.JNS151224

[7] Deletis V Fernandez-Conejero I Ulkatan S Methodology for intra-operative recording of the corticobulbar motor evoked potentials from cricothyroid muscles. Clin Neurophysiol 2011;122:1883–9. 21440494 10.1016/j.clinph.2011.02.018

[8] Ulkatan S Deletis V Fernandez-Conejero I Central or peripheral activations of the facial nerve? J Neurosurg 2007;106:519–20. author reply 520. 17367086 10.3171/jns.2007.106.3.519

[9] Simioni V Capone JG Sette E 89 Facial nerve monitoring during cerebellopontine angle surgery: our experience. Clini Neurophysiol 2013;124:e209.

[10] Holland NR Intraoperative electromyography. J Clin Neurophysiol 2002;19:444. 12477989 10.1097/00004691-200210000-00007

[11] Nanda A Chittiboina P Facial nerve monitoring in posterior fossa surgery. World Neurosurg 2013;80:e197–8. 22381285 10.1016/j.wneu.2011.12.072

[12] Liu SW Jiang W Zhang HQ Intraoperative neuromonitoring for removal of large vestibular schwannoma: facial nerve outcome and predictive factors. Clin Neurol Neurosurg 2015;133:83–9. 25867236 10.1016/j.clineuro.2015.03.016

[13] Goto T Muraoka H Kodama K Intraoperative monitoring of motor evoked potential for the facial nerve using a cranial peg-screw electrode and a “threshold-level” stimulation method. Skull Base 2010;20:429. 21772800 10.1055/s-0030-1261270 PMC3134811

[14] Dong CC Macdonald DB Akagami R Intraoperative facial motor evoked potential monitoring with transcranial electrical stimulation during skull base surgery. Clin Neurophysiol 2005;116:588–96. 15721072 10.1016/j.clinph.2004.09.013

[15] Sarnthein J Hejrati N Neidert MC Facial nerve motor evoked potentials during skull base surgery to monitor facial nerve function using the threshold-level method. Neurosurg Focus 2013;34:E7. 10.3171/2012.12.FOCUS12386 23451854

[16] Akagami R Dong CC Westerberg BD Localized transcranial electrical motor evoked potentials for monitoring cranial nerves in cranial base surgery. Neurosurgery 2005;57:78–85. 15987572 10.1227/01.neu.0000163486.93702.95

[17] Fukuda M Oishi M Hiraishi T Intraoperative facial nerve motor evoked potential monitoring during skull base surgery predicts long-term facial nerve function outcomes. Neurol Res 2011;33:578–82. 21708066 10.1179/016164110X12700393823697

[18] House JW Brackmann DE Facial nerve grading system. Laryngoscope 2010;93:1056–69. 10.1288/00005537-198308000-00016 6877014

[19] Calancie B Molano MR Alarm criteria for motor-evoked potentials: what's wrong with the “presence-or-absence” approach? Spine (Phila Pa 1976) 2008;33:406–14. 18277873 10.1097/BRS.0b013e3181642a2f

[20] Sarnthein J Bozinov O LP33: intraoperative monitoring of facial nerve motor evoked potentials in children. World Neurosurg 2014;125:786–94. 10.1016/j.wneu.2015.05.008 25986204

[21] Merton PA Morton HB Stimulation of the cerebral cortex in the intact human subject. Nature 1980;285:227. 7374773 10.1038/285227a0

[22] Deletis V Camargo AB Transcranial electrical motor evoked potential monitoring for brain tumor resection. Neurosurgery 2001;49:1075–80. 10.1097/00006123-200112000-00049 11859835

[23] Tsutsui S Yamada H Basic principles and recent trends of transcranial motor evoked potentials in intraoperative neurophysiologic monitoring. Neurol Med Chir 2016;56:451–6. 10.2176/nmc.ra.2015-0307 PMC4987444 26935781

[24] Acioly MA Liebsch M de Aguiar PH Facial nerve monitoring during cerebellopontine angle and skull base tumor surgery: a systematic review from description to current success on function prediction. World Neurosurg 2013;80:e271–300. 22120256 10.1016/j.wneu.2011.09.026

[25] Romstöck J Strauss C Fahlbusch R Continuous electromyography monitoring of motor cranial nerves during cerebellopontine angle surgery. J Neurosurg 2000;93:586–93. 11014536 10.3171/jns.2000.93.4.0586

[26] Matthies C Raslan F Schweitzer T Facial motor evoked potentials in cerebellopontine angle surgery: technique, pitfalls and predictive value. Clin Neurol Neurosurg 2011;113:872–9. 21798660 10.1016/j.clineuro.2011.06.011

[27] Macdonald DB Safety of intraoperative transcranial electrical stimulation motor evoked potential monitoring. J Clin Neurophysiol 2002;19:416–29. 12477987 10.1097/00004691-200210000-00005

